# Perceived Impact of Social Media on Panic Buying: An Online Cross-Sectional Survey in Iraqi Kurdistan

**DOI:** 10.3389/fpubh.2021.668153

**Published:** 2021-05-10

**Authors:** S.M. Yasir Arafat, Araz Ramazan Ahmad, Hersh Rasool Murad, Hardawan Mahmoud Kakashekh

**Affiliations:** ^1^Department of Psychiatry, Enam Medical College and Hospital, Dhaka, Bangladesh; ^2^Department of Administration, College of Humanities, University of Raparin, Ranya, Iraq; ^3^Department of International Relations & Diplomacy, Faculty of Administrative Sciences and Economics, Tishk International University, Erbil, Iraq; ^4^Public Relations and Marketing Department, Technical College of Administration, Sulaimani Polytechnic University (SPU), Sulaymaniyah, Iraq; ^5^Department of Media, College of Arts, Salahaddin University-Erbil, Erbil, Iraq

**Keywords:** panic buying, social media, COVID-19, stockpiling, Iraq

## Abstract

**Background:** Social media has an impact on panic buying by creating fear, disseminating pictures, and videos of people purchasing extra goods in a state of panic during the COVID-19 pandemic.

**Aim:** We aimed to evaluate the perceived impact of social media on panic buying behaviors in the Iraqi Kurdistan region.

**Methods:** This cross-sectional survey was conducted from October 10 to November 25, 2020. A self-reported instrument was designed in English and then translated to the Kurdish Language to conduct the study. It was disseminated through social media platforms (Facebook, Viber, and WhatsApp) and e-mail, *via* a link, and 466 responses were collected from social media users. The statistical computations were performed using SPSS version 21.

**Results:** The majority of respondents were male (62.2%), were <25 years old (43.9%), and had completed their bachelor's degree (53.9%), and most of the respondents (86.3%) used Facebook. Among the respondents, 42.1% were involved in panic buying, 32.8% of the respondents thought that social media platforms had an influence on panic buying, 86.1% of the respondents thought that social media should be sensible while reporting it, 88.4% thought that the reporting should be controlled, and 78.5% thought that photos of empty shelves should be avoided. There was a significant positive statistical correlation (*r* = 0.84) between social media use and panic buying among consumers during the COVID-19 pandemic in Iraqi Kurdistan (*p* = <0.001).

**Conclusion:** This research assessed how social media affects buying behavior, particularly in Iraq. Collective measures, such as sensible use and adequate media literacy, are needed to prevent such behaviors at least during public health emergencies.

## Introduction

The COVID-19 pandemic has disrupted the world economy and medical services, generating fear, panic, and vulnerability among billions of individuals. During the initial phase of COVID-19 spreading and mandatory lockdowns in numerous locations, panic buying (PB) has arisen as a common component of the COVID-19 outbreak (1). Store racks have been stripped bare across countries. Toiletries, frozen food, rice, beans, eggs, and bread are some basic goods that were regularly sold out as customers made hasty purchases and bought extra amounts (2). There are some logical explanations behind this behavior, such as the purchase of many tissues, which might be identified with the critical and pointless requirement of washing hands, or as an intelligent response to watching pictures of frenzy purchases in different nations (3–5). One study has proposed the causative model of PB, suggesting that there is usually an adverse stimulus such as disaster, war, or a pandemic (4). Subsequently, other factors such as media shape the initial response (4).

The panic behavior of buying toilet paper was one of the predominant, appalling, and stunning recordings that was viewed by people across the world on social media platforms. Numerous individuals have shared stories, pictures, and encounters on Twitter and Facebook which additionally furthered frenzy purchasing among different shoppers (6, 7). Mao (7) explains that social media has encouraged the utilization of some hashtags, for instance “#toilet-paper-gate” and “#toilet-paper-crisis,” which demonstrate shoppers' frenzied behavior during the COVID-19 pandemic. Scientists have reported that business exercises and buyer purchasing behaviors have been altered due to the COVID-19 pandemic and its controlling measures (8).

The Iraqi people have for the most part been in crisis since 1921, after the creation of Iraq. The political instability and economic situation have always been in turmoil (9). Therefore, Iraqis usually buy more goods and store them. However, since the emergence of COVID-19, the episodes of PB have been reported, particularly for some protective equipment such as gloves, masks, protective materials, food, and toilet paper. Along with the enduring conflict, the COVID-19 pandemic acts as a precipitating event for initiating PB as previous studies reported that crises, conflict, pandemics, health-care emergencies precipitate it (2–4).

Media has an important causative as well as the preventive role in PB (4, 10, 11). There are various forms of media: digital media platforms have connected all users through their phones, and computer networks connect users through computerized markets, internet banking, and shopping. Diverse web-based media platforms have emerged providing worldwide availability to clients (12). Ahmad & Murad (13) claim that posts on social media platforms in Iraq have had a significant impact on the psychological aspects of society, creating panic among people, particularly during the first months of the emergence and spread of COVID-19. Other studies have found that that there is a lack of comprehension of how online media can shape fear and customer reactions, triggering PB during the COVID-19 outbreak across the world (14, 15). However, the impact of social media on PB, particularly in influencing the consumers to buy more, has not been assessed in many countries including Iraq. As a conflict-prone region, the findings of this study would help to examine the prevention of PB during other crises like war. In this study, we aimed to evaluate the perceived impact of social media on PB behaviors in the Iraqi Kurdistan region.

## Materials and Methods

### Data Collection

We applied a cross-sectional quantitative survey method to gather data from social media users in Iraqi Kurdistan, using Google forms. The survey was conducted from October 10 to November 25, 2020, and was disseminated through social media platforms (Facebook, Viber, and WhatsApp) and e-mail with a link. The survey was created in the Kurdish language, and 466 responses were collected from the social media users. The random online questionnaire was aimed to determine the perceived impact of social media on PB among consumers in Iraqi Kurdistan. The scope of the survey and objectives of the research were made available to all the respondents. The link to the questionnaire was circulated to the respondents, and they were requested to respond. A summary containing the objectives and the procedures was displayed by clicking on the questionnaire link, followed by the mandatory consent form. Respondents who provided consent were able to complete the survey.

### Instrument

The instrument was designed by the authors, and the scale was made following a previous study on social media and panic in Iraq (13). The survey was designed in English and then translated to the Kurdish Language which is the formal language in Iraq. We prepared it in a clear and simple way with examples which was easy to understand the terms and concepts as panic buying is common among Iraqi people because of enduring crises. The questionnaire was composed of seven questions regarding the sociodemographic profile of the respondents (age, marital status, gender, education, number of people in a household, and monthly salary): three questions were regarding respondents' social media, and the remainder of the questions were regarding the impact of social media platforms on customers' fear of empty shelves. Cronbach's alpha was 0.92. We did not assess the mode of panic buying, frequency, and the name of the commodities.

### Statistical Analysis

All statistical computations were performed using SPSS version 21. Data were coded, tabulated, and presented in a descriptive form. The statistical procedure used to determine the results of the present study included Cronbach's alpha to test the reliability of the questionnaire, descriptive statistical data analysis (frequency, percentage, mean, standard deviation, coefficient of variance, and relative importance), and inferential data analysis: Pearson bivariate correlation and simple regression model.

### Ethical Statement

The study was conducted in compliance with the Helsinki declaration (1964). Permission was obtained from the University of Raparin, Iraq (ID Number: 7-29-319). Informed consent was obtained electronically while initiating the survey.

## Results

### Demography of the Respondents

The mean (±SD) age of the respondents was 29.63 (± 9.85) years. The majority of respondents were male (62.2%), were <25 years old (43.9%), had completed their bachelor's degree (53.9%), were employed full time (27.9%), and were single (52.4%) (Table 1). Most of the respondents (86.3%) used Facebook; 38.4% used social media for 2–4 h per day (Table 2). Among the respondents, 42.1% (*n* = 196) were involved in PB. Among the panic buyers, 35.2% (*n* = 69) were involved in the activity during the COVID-19 pandemic while 64.8% (*n* = 127) bought extra amounts during usual times.

**Table 1:** Demography of the respondents (*n* = 466).

**Variables**	***n* (%)**
**Sex**	
Male	290 (62.2)
Female	170 (36.5)
Prefer not to say	6 (1.3)
**Age in years**	
<25	204 (43.9)
25–35	150 (32.2)
36–45	77 (16.5)
46–55	26 (5.6)
More than 55	9 (1.9)
Mean ± SD	29.63 ± 9.85
**Highest level of education**	
High school	94 (20.2)
Bachelor	251 (53.9)
Masters	52 (11.2)
Ph.D. or higher	26 (5.6)
Prefer not to say	43 (9.2)
**Current employment status**	
Employed full-time	130 (27.9)
Employed part-time	90 (19.3)
Seeking opportunities	52 (11.2)
Retired	6 (1.3)
Prefer not to say	188 (40.3)
**Marital status**	
Single	244 (52.4)
Married	209 (44.8)
Prefer not to say	13 (2.8)
**Children**	
None	270 (57.9)
1	41 (8.8)
2–4	123 (26.4)
More than 4	14 (3)
Prefer not to say	18 (3.9)
**Monthly income**	
< $100	82 (17.6)
$100–$300	69 (14.8)
$300–$600	72 (15.5)
$600–$900	39 (8.4)
$900–$1200	78 (16.7)
more than $1200	126 (27)
**Total**	**466**

**Table 2:** Social media use profile of the respondents (*n* = 466).

**Variables**	***n* (%)**
**Skills of using social media platforms (subjective assessment)**	
Very good	9 (1.9)
Good	15 (3.2)
Medium	127 (27.3)
Bad	168 (36.1)
Very bad	147 (31.5)
**Social media platform**	
Facebook	402 (86.3)
Instagram	210 (45.1)
Snapchat	157 (33.7)
YouTube	182 (39.1)
Twitter	53 (11.4)
Viber	170 (36.5)
Line	5 (1.1)
WhatsApp	126 (27)
Telegram	118 (25.3)
**Duration of use of social media platforms in a day**	
Less than hour	10 (2.1)
1–2 h	118 (25.3)
2–4 h	179 (38.4)
4–6 h	92 (19.7)
More than 6 h	67 (14.4)
**Total**	**100.0**

### Perceived Impact of Social Media on PB

The perceived influence of social media on PB is displayed in Table 3. Only one-third (32.8%) of the respondents thought that social media platforms had an influence on creating PB, and one-sixth (16.1%) thought that the fear shown on social media can create PB (Table 3). The assessment of overall perceived influence revealed that less than one-third of the respondents were supposed to be influenced (Table 3). The majority (86.1%) of the respondents thought that social media should be sensible while reporting PB, 88.4% thought that the reporting should be controlled, and 78.5% thought that photos of empty shelves should be avoided (Table 4). There was a significant positive statistical correlation (*r* = 0.84) between usage of social media platforms and PB among consumers in Iraqi Kurdistan (*p* = <0.001) (Table 5). Table 6 indicates that the regression model predicted the dependent variable significantly. Here, the *p*-value (<0.001) was <0.05, which indicates that the regression model statistically predicts the outcome variable significantly (i.e., it is a good fit for the data). The R^2^ value indicates how much of the total variation in the dependent variable (PB) can be explained by the independent variable (social media). The R^2^ for this study was 0.7, indicating that 70% of the variance (of PB) is explained by the usage of social media.

**Table 3:** Perceived influence of social media on panic buying.

**Item**	**Totally disagree (*n*, %)**	**Disagree (*n*, %)**	**Neutral (*n*, %)**	**Agree (*n*, %)**	**Totally agree (*n*, %)**	**Mean ± S.D**
>Social media platforms have influence on creating panic buying.	18 (3.9)	244 (52.4)	51 (10.9)	119 (25.5)	34 (7.3)	2.8 ± 1.08
>Fear on social media drives to panic buying.	151 (32.4)	208 (44.6)	32 (6.9)	55 (11.8)	20 (4.3)	2.11 ± 1.11
>Spreading the fear of not having the products on social media leads to buying more things.	142 (30.5)	188 (40.3)	51 (10.9)	49 (10.5)	36 (7.7)	2.25 ± 1.21
>I panic when I saw the photos and video of empty shelves of essential products on social media.	186 (39.9)	128 (27.5)	44 (9.4)	39 (8.4)	69 (14.8)	2.3 ± 1.44
>The feeling of uncertainty during emergency influences my buying habits.	118 (25.3)	199 (42.7)	31 (6.7)	73 (15.7)	45 (9.7)	2.42 ± 1.28

**Table 4:** Preventive aspects of panic buying on social media.

**Questions**	**No (*n*, %)**	**Don't know (*n*, %)**	**Yes (*n*, %)**	**Mean ± S.D**
Do you think that social media reports should be sensible while reporting panic buying?	25 (5.4)	40 (8.6)	401 (86.1)	2.8 ± 0.54
Do you think that social media reports should be controlled while reporting panic buying?	33 (7.1)	21 (4.5)	412 (88.4)	2.81 ± 0.51
Do you think that photos of empty shelves should be avoided while social media reporting panic buying?	39 (8.4)	61 (13.1)	266 (78.5)	2.7 ± 0.61
Sum	97 (6.94)	122 (8.7)	1179 (84.3)	2.77 ± 0.55

**Table 5:** Association between social media and panic buying.

		**Social media platforms**	**Panic buying**
Social media platforms	Correlation	1	0.838
	Sig.		0.000
Panic buying	Correlation	0.838	1
	Sig.	0.000	

**Table 6:** The effects of social media platforms on panic buying among consumers during the COVID-19 pandemic in Iraqi Kurdistan.

**Model**	**Coefficients**				**Model summary**			**ANOVA**	
	**Unstandardized coefficients**		***T*-test**	**Sig**.	**R**	**R^**2**^**	**Adjust R^**2**^**	**F Test**	**Sig**.
	**B**	**Std. error**							
Constant	0.522	0.042	8.376	0.00	0.838	0.702	0.701	1093.15	0.00
Social media	0.832	0.025	33.06	0.00					

## Discussion

Panic buying received the focus of researchers during the COVID-19 pandemic (16). Newer studies exploring its aspects are being published (16, 17). Media plays a bidirectional role in the case of PB. Hypothetically, it can increase fear by disseminating pictures of the empty shelves and rumors of a shortage of supply (4, 10, 11, 15). Previous studies revealed that mass media and social media have a role in disseminating fear and rumors, which in turn increases erratic behavior like PB (10, 12, 18, 19). Furthermore, it has been reported that fake news on social media demonstrated a strong positive impact on impulse buying (18–20). In contrast, mass media and social media can minimize PB by reducing public tension and threat and assuring the public that there is a sufficient supply of good. However, the role of social media in PB is yet to be established by empirical studies. We aimed to assess whether social media has a role on PB behavior in Iraqi Kurdistan.

### Main Findings of the Study

The study revealed that Facebook was the most commonly used platform, with more than one-third of the respondents spending 2–4 h per day on social media (Table 2). Further, 42.1% (*n* = 196) were involved in PB and 35.2% (*n* = 69) of the respondents exhibited this behavior during the COVID-19 pandemic.

The perceived influence of social media as measured by the responses was identified in less than one-third of the respondents (Table 3). However, the respondents thought that social media should be monitored as a controlling measure. Furthermore, we found a significant positive statistical correlation between usage of social media platforms and panic buying among consumers (Table 5). A previous study following similar methods had similar findings regarding social media platforms. The study measured the impact of social media on panic and reported that the spread of panic and anxiety was significantly related to self-reported social media use (13). It was revealed that social media can help promote social exchange, resulting in proactive activities in the form of consumer PB (14). Additionally, other studies reported that PB was more common among people who were more worried about the COVID-19 pandemic and that social media possibly played a role in disseminating the worry, fear, and anxiety by showing images of empty shelves, which in turn produced a sense of scarcity and short supply (19–21). Studies from different settings have also reported that rumors regarding the shortage of supplies increased PB (22–24).

Owing to the development of new technology and the progress of new forms of media, people spend more time on social media than before. Within seconds, new information, news, pictures, and videos can be posted to millions of people. The study shows that social media has an impact in creating PB during the COVID-19 pandemic and that social media posts spread panic which caused people to buy more products than needed.

### Implications of the Study Results

During any public health emergency, people get anxious because of uncertainly and look for information. For this purpose, social media is an easily available option. However, it is difficult to distinguish between real and false information on social media. False information and rumors can increase consumer anxiety. As a result, it is possible that social media could exacerbate feelings of helplessness leading to emotional distress. To avoid information contamination, it might be appropriate to prohibit people from sharing pictures of empty shelves. Facebook has the ability to block posts based on their content. It is critical to establish an enduring collaboration between the region's health practitioners and for media experts to ensure that only helpful characteristics of reporting of PB are disseminated (1, 10, 11).

### Challenges of Assessing the Relationship of Social Media and Panic Buying

Estimation of a precise relationship between several aspects of social media and PB is challenging for several reasons. Firstly, social media is supposed to have a mediating effect while other major factors (4, 17). Secondly, PB appears suddenly during crises, occurs in irregular bouts, and is difficult to predict (25). Thirdly, as an emerging topic with fundamental challenges to study, there is an extreme dearth of empirical studies to ascertain such associations (25). Fortunately, newer studies are coming out even though further studies are warranted (14, 15, 19). Fourthly, there are numerous confounding variables such as socio-demography, economic construct, previous experience, and the public health crisis itself. Fifthly, there is no established guideline for social media use and controlling the misuse. Finally, social media gets the poor attention of the public health agency during a pandemic in comparison to the pandemic itself.

### Strengths of the Study

This is the first study assessing the impact of social media on PB in a conflict-prone area. As responses were collected through online platforms and a self-reporting instrument, the chances of having conformity biases are lower.

### Limitations

This study has some limitations related to data collection, reading comments, and obtaining feedback from respondents on social media reports about empty shelves in shops and markets. Quantification of the PB was not performed. The instrument was self-reporting, and responses were collected via online platforms, which may produce recall biases. There is a potential chance of biases in understanding the meaning and terminology as the PB phenomenon was not explained explicitly to all the respondents. The instrument was not psychometrically tested before the study. Collecting data during the pandemic was not an easy process, particularly with people who had been affected by the panic of COVID-19. Participants were not assessed or screened for any psychological disorders and other confounding variables affecting social media use have not been considered.

### Conclusion

This research assessed how social media affects buying behavior particularly in Iraq. Collective measures such as sensible use and adequate media literacy are needed to prevent the behaviors, at least during public health emergencies. The regulatory bodies and social media companies could implement new regulations on social media platforms during crises and pandemics to controlling PB. It could be used as the universal and primary prevention strategies while considering the prevention of PB during the forthcoming disasters. Culturally appropriate interventions should be developed and tested as PB has been noticed during crises and public health emergencies.

## Data Availability Statement

The raw data supporting the conclusions of this article will be provided on request to the corresponding author.

## Ethics Statement

The study was conducted complying with the Helsinki declaration (1964). Ethical clearance was obtained from University of Raparin, Iraq (ID Number: 7-29-319).

## Author Contributions

SA contributed to the concept, design, and writing. AA, HM, and HK contributed to data collection and writing. All the authors contributed to manuscript writing, revision, reading, and approval of the submitted version.

## Conflict of Interest

The authors declare that the research was conducted in the absence of any commercial or financial relationships that could be construed as a potential conflict of interest.
